# Serum IL-32γ as a biomarker for remission assessment in systemic lupus erythematosus

**DOI:** 10.3389/fimmu.2026.1915750

**Published:** 2026-08-05

**Authors:** Oh Chan Kwon, Min-Chan Park, Yong-Gil Kim

**Affiliations:** 1 Division of Rheumatology, Department of Internal Medicine, Gangnam Severance Hospital, Yonsei University College of Medicine, Seoul, Republic of Korea; 2 Division of Rheumatology, Department of Internal Medicine, Asan Medical Center, University of Ulsan College of Medicine, Seoul, Republic of Korea

**Keywords:** biomarker, IL-32γ, lupus low disease activity state, remission, systemic lupus erythematosus

## Abstract

**Background:**

To investigate the potential of serum interleukin (IL)-32γ levels as a biomarker for assessing lupus low disease activity state (LLDAS) and remission, the targets of a treat-to-target strategy, in patients with systemic lupus erythematosus (SLE).

**Methods:**

We analyzed sera from 40 patients with SLE. Serum IL-32γ levels were measured using an enzyme-linked immunosorbent assay, alongside conventional serologic markers (C3, C4, anti-double-stranded DNA antibody). Disease status was classified according to LLDAS, definition of remission in SLE (DORIS) remission, and glucocorticoid (GC)-free remission. Correlations with SLE disease activity index 2000 (SLEDAI-2K) were examined using Spearman’s rank correlation coefficient, and receiver operating characteristic (ROC) curve analyses were performed to evaluate discriminative accuracy for disease state.

**Results:**

Serum IL-32γ levels correlated significantly with SLEDAI-2K (rho=0.401, p=0.010), as did the conventional serologic markers. In ROC analyses, IL-32γ was statistically significant in discriminating LLDAS (area under the curve [AUC]=0.728, p=0.014), similar to the other conventional serologic markers. However, for more stringent definitions, IL-32γ was the only serologic marker that retained statistical significance (DORIS remission: AUC = 0.785, p=0.014; and GC-free remission: AUC = 0.831, p=0.007).

**Conclusion:**

Serum IL-32γ is significantly associated with overall disease activity and shows promising discriminatory ability for disease states in SLE. Notably, it retains discriminative capacity in stringent remission definitions, underscoring its potential as a novel biomarker. These findings suggest that IL-32γ could complement existing tools in treat-to-target strategies, offering a simple serologic marker to guide therapy toward the ultimate goal of GC-free remission.

## Introduction

Systemic lupus erythematosus (SLE) is a complex autoimmune disease characterized by multisystem involvement, fluctuating disease activity, and significant morbidity (1, 2). A treat-to-target approach, aiming to achieve remission, or at least the lupus low disease activity state (LLDAS), is now widely adopted in SLE management (3, 4). Achievement of LLDAS or remission has been consistently shown to lower the risk of organ damage accrual, disease flare, impaired health-related quality of life, and mortality (5–8). Successful implementation of a treat-to-target strategy requires accurate assessment of the disease state, particularly the attainment of LLDAS or remission. Both the LLDAS and 2021 definition of remission in SLE (DORIS) integrate multiple parameters, including the SLE Disease Activity Index 2000 (SLEDAI-2K), physician global assessment (PGA), and glucocorticoid (GC) dosage to determine disease activity (9, 10). Moreover, assessment of SLEDAI-2K requires evaluation of diverse clinical manifestations, and serologic markers such as C3, C4, and anti-double-stranded DNA (dsDNA) antibody (Ab), as well as complete blood count and urinalysis results (11). Given the complexity of assessing LLDAS or remission, there remains a clear need for a simple biomarker to facilitate evaluation of these disease states.

Interleukin (IL)-32, previously referred to as Natural Killer Cell Transcript 4, is an important cytokine involved in both innate and adaptive immune responses (12). Multiple isoforms of IL-32 have been identified, with IL-32γ demonstrating the most potent biologic activity (13). Studies have reported higher serum levels of IL-32γ in patients with SLE than in healthy controls, suggesting its possible pathogenic role in SLE (14, 15). Given its role in amplifying immune responses (12) and its elevated serum levels in patients with SLE (14, 15), serum IL-32γ may serve as a promising biomarker for assessing LLDAS or remission.

On this basis, we investigated the potential utility of serum IL-32γ levels as a biomarker for detecting LLDAS and remission status in patients with SLE.

## Materials and methods

### Participants

Sera from patients with SLE were collected at a tertiary referral hospital in Seoul, South Korea. All patients met the 2019 European League Against Rheumatism/American College of Rheumatology classification criteria for SLE (16). The following covariates were obtained at the time of serum collection: age, sex, disease duration, clinical manifestations of SLE, SLEDAI-2K, PGA, erythrocyte sedimentation rate (ESR), C-reactive protein (CRP), serum C3 and C4 levels, serum anti-dsDNA Ab titer, serum positivity for anti-Sm Ab, anti-Ro Ab, anti-La Ab, and anti-U1RNP Ab; presence of urine red blood cells (> 5/high-power field [HPF]) and white blood cells (> 5/HPF); urine protein-to-creatinine ratio, and medication use, including current use of hydroxychloroquine and immunosuppressants, as well as daily prednisolone dose.

This study was approved by the Institutional Review Board (IRB) of Gangnam Severance Hospital (IRB No. 3-2020-0253), and informed consent was obtained from all participants.

### Measurement of serum IL-32γ levels

Serum IL-32γ levels were measured using a commercially available enzyme-linked immunosorbent assay kit (AFG Scientific, Northbrook, IL, USA) according to the manufacturer’s instructions. The lower limit of detection was 10 pg/mL, and the analytical range was 50–1,000 pg/mL. All samples were assayed in duplicate across two independent assay plates, and the mean values were used for subsequent analysis. The inter-assay coefficient of variation was 10.7%.

### Definition of LLDAS and remission

LLDAS was defined as a SLEDAI-2K score ≤ 4, a PGA score ≤ 1, and a prednisolone dose ≤ 7.5 mg/day (9). Remission was defined according to the 2021 DORIS criteria (clinical SLEDAI-2K score = 0, PGA score < 0.5, and prednisolone dose ≤ 5 mg/day) (10). In addition, a stricter definition of remission with a lower GC threshold—GC-free remission—was also applied, defined as clinical SLEDAI-2K score = 0, PGA score < 0.5, and prednisolone dose = 0 mg/day (8). Compared with LLDAS, which permits limited residual disease activity and prednisolone use up to 7.5 mg/day, DORIS remission is a more stringent definition because it requires the absence of clinical disease activity, a lower PGA threshold, and a lower allowable prednisolone dose. GC-free remission is even more stringent because it requires fulfillment of the clinical criteria for DORIS remission without any GC use.

### Statistical analysis

Continuous and categorical variables were expressed as median (interquartile range) and number (%), respectively. The correlation between serum IL-32γ levels and SLEDAI-2K was assessed using Spearman’s rank correlation coefficient. Correlations between conventional serologic markers (C3, C4, and anti-dsDNA Ab) and SLEDAI-2K were also evaluated for comparison. Mann–Whitney U test was used to compare serum C3, C4, anti-dsDNA Ab, and IL-32γ levels between groups. Univariable and multivariable logistic regression analyses were performed to evaluate the associations between serum IL-32γ levels and LLDAS, DORIS remission, and GC-free remission. Given the limited number of events, the multivariable models were restricted to serum IL-32γ and only one potential confounder to avoid overfitting. Hydroxychloroquine use, which is well established to be associated with the maintenance of remission (17), was selected as a clinically relevant potential confounder. Receiver operating characteristic (ROC) curve analysis was performed to assess the ability of serum C3, C4, anti-dsDNA Ab, and IL-32γ levels to discriminate disease states. The area under the curve (AUC) and 95% confidence interval (CI) for each parameter were estimated. The optimal cut-off of serum IL-32γ levels for discriminating GC-free remission, the most stringent definition, was determined as the point at which the Youden index was maximum. Using this cut-off, the sensitivity, specificity, positive predictive value (PPV), and negative predictive value (NPV) for discriminating GC-free remission were estimated with their respective 95% CI. The primary hypothesis was that serum IL-32γ levels are associated with remission status in SLE, whereas conventional serologic markers were included only as contextual reference markers rather than as multiple independent biomarkers of equal interest. Accordingly, correction for multiple comparisons was not applied. A p-value of < 0.05 was considered statistically significant. All analyses were conducted using GraphPad Prism version 10 (GraphPad Software, La Jolla, CA, USA).

## Results

### Participant characteristics

A total of 40 patients with SLE were included. Their characteristics at the time of serum collection are summarized in **Table 1**. The median age was 42.5 (27.5–49.0) years, and 38 (95.0%) were female. The median disease duration was 36.1 (20.7–60.7) months. Among SLE manifestations, mucocutaneous involvement (37.5%) was most common, followed by musculoskeletal manifestations (25.0%) and lupus nephritis (20.0%). The median values of SLEDAI-2K, clinical SLEDAI-2K, PGA, C3, C4, anti-dsDNA Ab, and prednisolone dose were 4.5 (2.0–10.0), 4.0 (0.0–6.0), 1.0 (1.0–2.0), 84.8 (68.9–101.0) mg/dL, 13.7 (9.9–22.7) mg/dL, 12.0 (3.0–62.0) IU/mL, and 2.5 (0.0–6.9) mg/day, respectively. The median serum IL-32γ level was 132.6 (116.1–152.2) pg/mL. Of the 40 patients, 19 (47.5%), 8 (20.0%), and 7 (17.5%) met the criteria for LLDAS, DORIS remission, and GC-free remission, respectively.

**Table 1:** Baseline characteristics of the 40 patients with SLE.

Characteristics	N = 40
Age, years, median (IQR)	42.5 (27.5–49.0)
Female sex, n (%)	38 (95.0)
Disease duration, months, median (IQR)	36.1 (20.7–60.7)
SLE manifestations, n (%)	
Mucocutaneous	15 (37.5)
Musculoskeletal	10 (25.0)
Lupus nephritis	8 (20.0)
Serositis	1 (2.5)
Neuropsychiatric	2 (5.0)
Hematologic	7 (17.5)
SLEDAI-2K, median (IQR)	4.5 (2.0–10.0)
Clinical SLEDAI-2K, median (IQR)	4.0 (0.0–6.0)
PGA, median (IQR)	1.0 (1.0–2.0)
ESR, mm/hr, median (IQR)	16.5 (8.3–23.0)
CRP, mg/L, median (IQR)	0.7 (0.2–1.8)
Serum C3, mg/dL, median (IQR)	84.8 (68.9–101.0)
Serum C4, mg/dL, median (IQR)	13.7 (9.9–22.7)
Serum anti-dsDNA Ab, IU/mL, median (IQR)	12.0 (3.0–62.0)
Serum anti-Sm Ab positive, n (%)	19 (47.5)
Serum anti-Ro Ab positive, n (%)	30 (75.0)
Serum anti-La Ab positive, n (%)	12 (30.0)
Serum anti-U1RNP Ab positive, n (%)	17 (42.5)
Urine RBC > 5/HPF, n (%)	2 (5.0)
Urine WBC > 5/HPF, n (%)	9 (22.5)
Urine protein/creatinine ratio, mg/g, median (IQR)	105.4 (69.5–140.0)
Serum IL-32γ, pg/mL, median (IQR)	132.6 (116.1–152.2)
Medications	
Hydroxychloroquine, n (%)	36 (90.0)
Cyclophosphamide, n (%)	0 (0.0)
Mycophenolate mofetil, n (%)	9 (22.5)
Azathioprine, n (%)	4 (10.0)
Methotrexate, n (%)	5 (12.5)
Tacrolimus, n (%)	7 (17.5)
Belimumab, n (%)	2 (5.0)
Rituximab, n (%)	0 (0.0)
GC, mg/day^*^, median (IQR)	2.5 (0.0–6.9)
LLDAS, n (%)	19 (47.5)
DORIS remission, n (%)	8 (20.0)
GC-free remission, n (%)	7 (17.5)

^*^ Prednisolone or its equivalent.

SLE, systemic lupus erythematosus; IQR, interquartile range; SLEDAI-2K, systemic lupus erythematosus disease activity index 2000; PGA, physician global assessment; ESR, erythrocyte sedimentation rate; CRP, C-reactive protein; dsDNA, double-stranded DNA; Ab, antibody; RBC, red blood cell; HPF, high power field; WBC, white blood cell; IL, interleukin; GC, glucocorticoid; LLDAS, lupus low disease activity state; DORIS, definition of remission in systemic lupus erythematosus.

### Correlation between serum C3, C4, anti-dsDNA Ab, and IL-32γ levels and SLEDAI-2K

**Figure 1:**
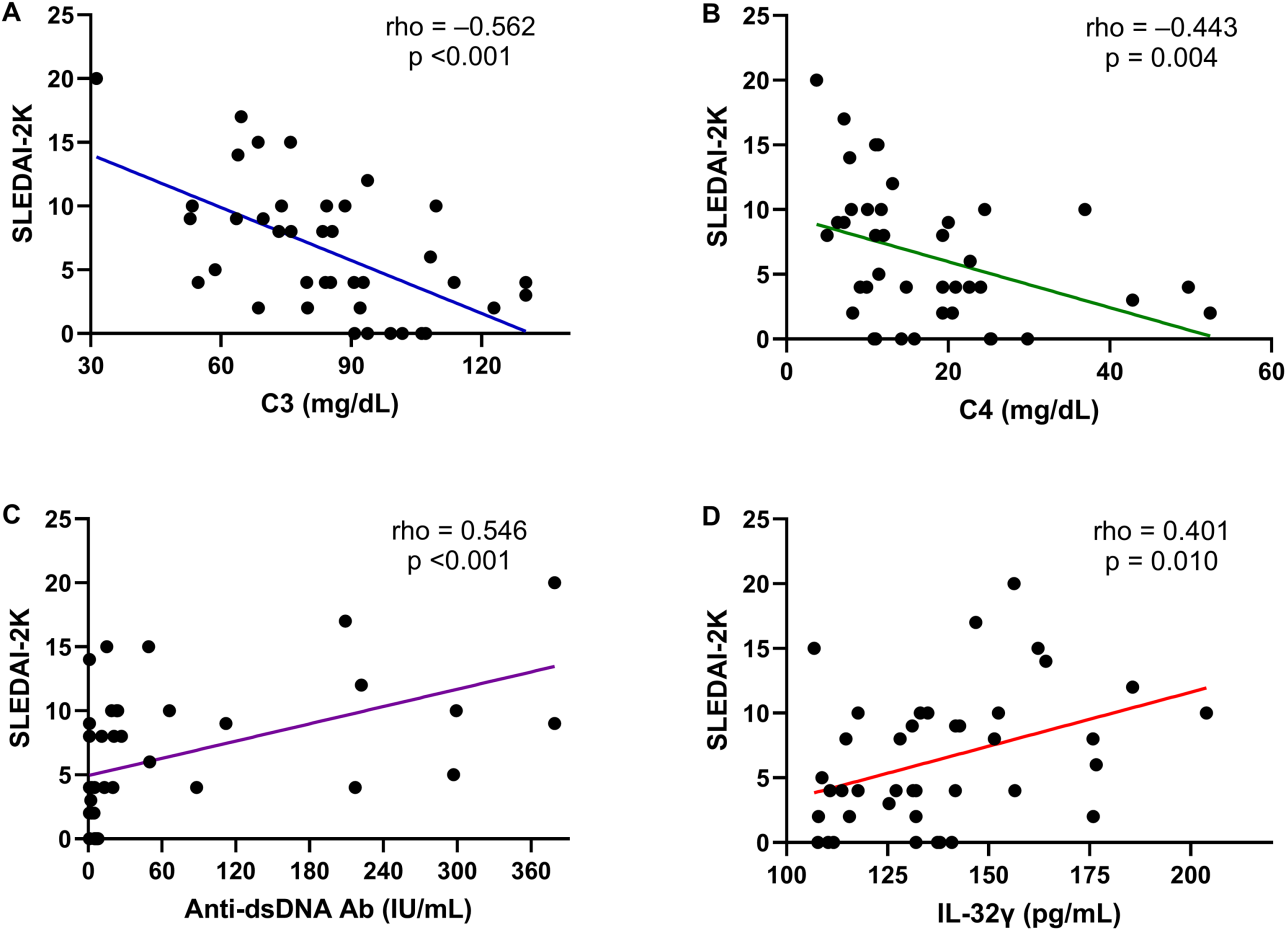
Scatter plots with correlation lines depicting correlation between SLEDAI-2K and **(A)** serum C3, **(B)** serum C4, **(C)** serum anti-dsDNA Ab, and **(D)** serum IL-32γ levels. SLEDAI-2K, systemic lupus erythematosus disease activity index 2000; dsDNA Ab, double-stranded DNA antibody; IL, interleukin.

As expected, conventional serologic markers, including C3 (**Figure 1A**: rho = –0.562, p < 0.001), C4 (**Figure 1B**: rho = –0.443, p = 0.004), and anti-dsDNA Ab (**Figure 1C**: rho = 0.546, p <0.001), showed significant correlations with SLEDAI-2K. To further explore whether serum IL-32γ could serve as a potential biomarker for assessing LLDAS and remission state, we analyzed its correlation with SLEDAI-2K, and found that IL-32γ was also significantly correlated with SLEDAI-2K (**Figure 1D**: rho = 0.401, p = 0.010).

### Comparison of serum C3, C4, anti-dsDNA Ab, and IL-32γ levels according to disease status

**Figure 2:**
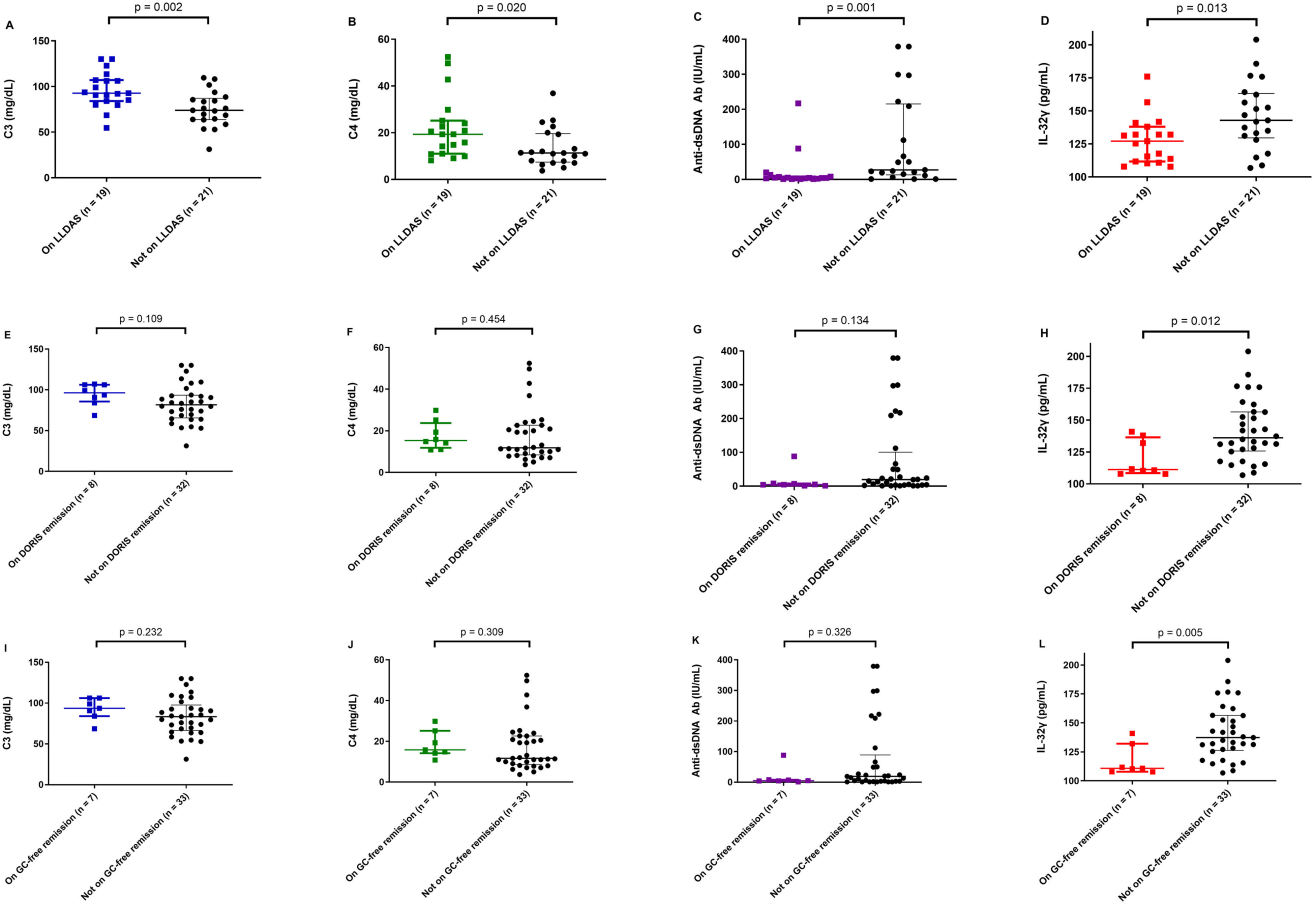
Scatter plots with median and interquartile range for comparison of **(A)** serum C3, **(B)** serum C4, **(C)** serum anti-dsDNA Ab, and **(D)** serum IL-32γ levels according to LLDAS; comparison of **(E)** serum C3, **(F)** serum C4, **(G)** serum anti-dsDNA Ab, and **(H)** serum IL-32γ levels according to DORIS remission; and comparison of **(I)** serum C3, **(J)** serum C4, **(K)** serum anti-dsDNA Ab, and **(L)** serum IL-32γ levels according to GC-free remission. Bars indicate median and interquartile range. dsDNA Ab, double-stranded DNA antibody; IL, interleukin; LLDAS, lupus low disease activity state; DORIS, definition of remission in systemic lupus erythematosus; GC, glucocorticoid.

**Figure 2** presents the comparison of serum C3, C4, anti-dsDNA Ab, and IL-32γ levels according to disease status. Serum C3 (**Figure 2A**: 92.7 [84.6–106.7] mg/dL vs. 73.9 [63.9–85.6] mg/dL, p = 0.002) and C4 levels (**Figure 2B**: 19.3 [12.6–24.6] mg/dL vs. 11.3 [7.8–19.3] mg/dL, p = 0.020) were significantly higher in patients who were on LLDAS than in those who were not. Conversely, serum anti-dsDNA Ab (**Figure 2C**: 4.0 [2.5–7.5] IU/mL vs. 27.0 [15.0–209.0] IU/mL, p = 0.001) and IL-32γ levels (**Figure 2D**: 127.1 [112.7–135.0] pg/mL vs. 142.8 [131.1–162.2] pg/mL, p = 0.013) were significantly lower in patients who achieved LLDAS than in those who did not.

In the comparison between patients who were in DORIS remission and those who were not, serum C3 (**Figure 2E**: 96.4 [87.4–106.2] mg/dL vs. 81.7 [66.6–93.2] mg/dL, p = 0.109), C4 (**Figure 2F**: 15.3 [12.6–22.3] mg/dL vs. 11.9 [8.7–22.7] mg/dL, p = 0.454), and anti-dsDNA Ab levels (**Figure 2G**: 4.8 [2.5–7.5] IU/mL vs. 19.5 [3.0–89.0] IU/mL, p = 0.134) did not differ significantly between the two groups. In contrast, serum IL-32γ levels (**Figure 2H**: 111.2 [109.1–135.0] pg/mL vs. 136.2 [126.2–156.4] pg/mL, p = 0.012) were significantly lower in patients who were in DORIS remission than in those who were not.

Similarly, serum C3 (**Figure 2I**: 93.7 [87.4–102.6] mg/dL vs. 83.4 [68.5–93.7] mg/dL, p = 0.232), C4 (**Figure 2J**: 15.8 [14.5–22.3] mg/dL vs. 11.7 [9.1–22.6] mg/dL, p = 0.309), and anti-dsDNA Ab levels (**Figure 2K**: 5.0 [4.3–7.5] IU/mL vs. 19.0 [3.0–66.0] IU/mL, p = 0.326) did not differ significantly between patients who were in GC-free remission and those who were not, whereas serum IL-32γ levels (**Figure 2L**: 110.8 [109.1–121.9] pg/mL vs. 137.4 [127.1–156.3] pg/mL, p = 0.005) were significantly lower in patients who were in GC-free remission than in those who were not.

### Association of IL-32γ and disease status

In the univariable logistic regression analyses, higher serum IL-32γ levels were associated with lower odds of being in LLDAS (unadjusted odds ratio [OR] = 0.961 [95% CI = 0.928–0.994], p = 0.022), DORIS remission (unadjusted OR = 0.936 [95% CI = 0.882–0.994], p = 0.031), and GC-free remission (unadjusted OR = 0.918 [95% CI = 0.853–0.989], p = 0.025). Furthermore, after adjusting for hydroxychloroquine use, higher serum IL-32γ levels remained significantly associated with lower odds of being in LLDAS (adjusted OR = 0.964 [95% CI = 0.932–0.998], p = 0.035), DORIS remission (adjusted OR = 0.941 [95% CI = 0.888–0.998], p = 0.043), and GC-free remission (adjusted OR = 0.924 [95% CI = 0.858–0.994], p = 0.035) (**Table 2**).

**Table 2:** Association between Serum IL-32γ and disease status.

Disease status	Univariable analysis		Multivariable analysis*	
	Unadjusted OR (95% CI)	P value	Adjusted OR (95% CI)	P value
LLDAS	0.961 (0.928–0.994)	0.022	0.964 (0.932–0.998)	0.035
DORIS remission	0.936 (0.882–0.994)	0.031	0.941 (0.888–0.998)	0.043
GC-free remission	0.918 (0.853–0.989)	0.025	0.924 (0.858–0.994)	0.035

*Adjusted for hydroxychloroquine use.

ORs are presented per 1 pg/mL increase in serum IL-32γ levels.

IL, interleukin; OR, odds ratio; CI, confidence interval; LLDAS, lupus low disease activity state; DORIS, definition of remission in systemic lupus erythematosus; GC, glucocorticoid.

### Accuracy in discriminating LLDAS and remission

**Figure 3:**
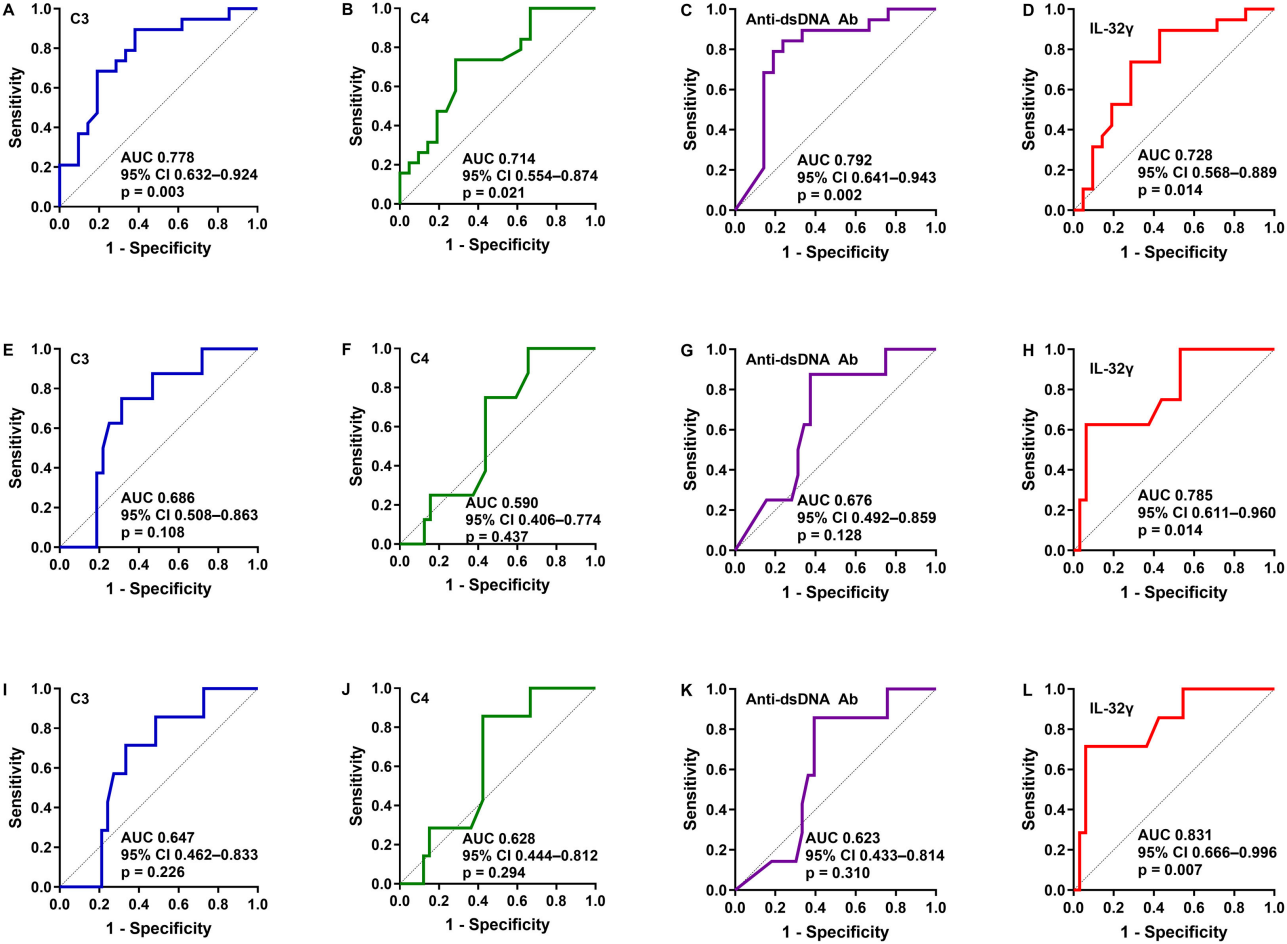
Receiver operating characteristic curves showing accuracy of **(A)** serum C3, **(B)** serum C4, **(C)** serum anti-dsDNA Ab, and **(D)** serum IL-32γ levels for assessing LLDAS; accuracy of **(E)** serum C3, **(F)** serum C4, **(G)** serum anti-dsDNA Ab, and **(H)** serum IL-32γ levels for assessing DORIS remission; and accuracy of **(I)** serum C3, **(J)** serum C4, **(K)** serum anti-dsDNA Ab, and **(L)** serum IL-32γ levels for assessing GC-free remission. dsDNA Ab, double-stranded DNA antibody; IL, interleukin; LLDAS, lupus low disease activity state; DORIS, definition of remission in systemic lupus erythematosus; GC, glucocorticoid; AUC, area under the curve; CI, confidence interval.

In the ROC analysis, serum C3 (**Figure 3A**: AUC = 0.778 [95% CI = 0.632–0.924], p = 0.003), C4 (**Figure 3B**: AUC = 0.714 [95% CI = 0.554–0.874], p = 0.021), anti-dsDNA Ab (**Figure 3C**: AUC = 0.792 [95% CI = 0.641–0.943], p = 0.002), and IL-32γ levels (**Figure 3D**: AUC = 0.728 [95% CI = 0.568–0.889], p = 0.014) were statistically significant for discriminating LLDAS.

For discriminating DORIS remission, serum C3 (**Figure 3E**: AUC = 0.686 [95% CI = 0.508–0.863], p = 0.108), C4 (**Figure 3F**: AUC = 0.590 [95% CI = 0.406–0.774], p = 0.437), and anti-dsDNA Ab levels (**Figure 3G**: AUC = 0.676 [95% CI = 0.492–0.859], p = 0.128) were not statistically significant, whereas serum IL-32γ levels (**Figure 3H**: AUC = 0.785 [95% CI = 0.611–0.960], p = 0.014) were statistically significant and showed good accuracy.

Similarly, serum C3 (**Figure 3I**: AUC = 0.647 [95% CI = 0.462–0.833], p = 0.226), C4 (**Figure 3J**: AUC = 0.628 [95% CI = 0.444–0.812], p = 0.294), and anti-dsDNA Ab levels (**Figure 3K**: AUC = 0.623 [95% CI = 0.433–0.814], p = 0.310) were not statistically significant, while serum IL-32γ (**Figure 3L**: AUC = 0.831 [95% CI = 0.666–0.996], p = 0.007) was the only serologic marker that was statistically significant in discriminating a stricter remission (i.e., GC-free remission). The optimal cut-off value of serum IL-32γ that best discriminated GC-free remission, determined by the point at which the Youden index was maximal, was 112.7 pg/mL. Using this cut-off, the sensitivity, specificity, PPV, and NPV for discriminating GC-free remission were 0.714 (95% CI 0.290–0.963), 0.939 (95% CI 0.798–0.993), 0.714 (95% CI 0.376–0.912), and 0.939 (95% CI 0.827–0.981), respectively.

## Discussion

In this study, we investigated the potential utility of serum IL-32γ as a biomarker for evaluating disease quiescence in patients with SLE, particularly regarding treat-to-target endpoints such as LLDAS and remission. Beyond assessing its association with disease activity alone, we specifically evaluated whether serum IL-32γ could discriminate clinically relevant remission states. Our findings demonstrated that serum IL-32γ levels were significantly correlated with SLEDAI-2K and were consistently lower in patients achieving LLDAS, DORIS remission, and GC-free remission. Notably, serum IL-32γ levels accurately identified patients in DORIS remission and GC-free remission, whereas conventional serologic markers, including C3, C4, and anti-dsDNA Ab, did not. The focus on remission assessment represents an important novelty of our study. These results highlight the potential role of IL-32γ as a serologic biomarker for assessing remission states in SLE.

Conventional serologic markers, including C3 (rho = –0.562, p < 0.001), C4 (rho = –0.443, p = 0.004), and anti-dsDNA Ab (rho = 0.546, p < 0.001), which are components of the SLEDAI-2K, showed significant correlation with SLEDAI-2K as expected. Moreover, serum IL-32γ levels (rho = 0.401, p = 0.010) were also significantly correlated with SLEDAI-2K. Although the absolute value of Spearman’s rho for IL-32γ was lower than that of C3, C4, and anti-dsDNA Ab, considering that IL-32γ is not included in the scoring system, its significant correlation with overall disease activity suggests its potential value as an additional biomarker for assessing disease activity.

When evaluating disease states defined by LLDAS, DORIS remission, and GC-free remission, the discriminative capacity of IL-32γ was distinct. For LLDAS, serum C3 (AUC = 0.778), C4 (AUC = 0.714), anti-dsDNA Ab (AUC = 0.792), and IL-32γ (AUC = 0.728) were all statistically significant, showing comparable discriminative accuracy. Since the definition of LLDAS allows for a certain degree of residual serologic or clinical activity (SLEDAI-2K ≤ 4), multiple serologic markers, including C3, C4, and anti-dsDNA Ab, may demonstrate comparable utility in distinguishing patients who achieve this state. However, in assessing DORIS remission and GC-free remission states, definitions capturing a deeper state of disease quiescence, IL-32γ (DORIS remission: AUC = 0.785; GC-free remission: AUC = 0.831) was the only marker that consistently showed statistically significant discriminative accuracy.

Notably, the discriminative accuracy of IL-32γ increased as more stringent definitions of remission were applied, suggesting that IL-32γ may be particularly suited for identifying patients in the deepest states of immunologic quiescence. This finding is clinically relevant, as accumulating evidence indicates that achieving GC-free remission is associated with improved long-term outcomes, including reduced risk of damage accrual and mortality, and has been suggested as a more desirable treatment target in SLE (8). Therefore, the ability of IL-32γ to discriminate patients in such stringent disease states highlights its potential as a clinically meaningful biomarker that could complement existing indices and guide therapeutic adjustments in SLE. Specifically, serum IL-32γ may be particularly informative in patients whose conventional serologic markers have normalized, a situation in which C3, C4, and anti-dsDNA Ab may not adequately reflect the depth of remission. In such cases, IL-32γ could provide complementary information to support the assessment of the depth of remission and help identify patients who may require closer monitoring.

The biological plausibility of IL-32γ as a biomarker for remission in SLE may be considered in the context of interferon (IFN) signatures. IFN-regulated gene expression in SLE is complex, comprising both type I and type II IFN signatures (18–22). Type I IFN-regulated genes are persistently upregulated in patients with SLE, regardless of disease activity, whereas type II IFN–regulated genes are preferentially upregulated in patients with high disease activity, and fluctuate over time in association with disease activity (23). Importantly, IL-32 has been reported to induce IL-12, a key cytokine that stimulates type II IFN (IFN-γ) production (24). Indeed, IL-32 has been shown to closely associate with type II IFN-driven immune responses in other diseases. For example, in giant cell arteritis, IL-32 was markedly upregulated in inflamed arterial tissues and strongly co-expressed with Th1 cytokines such as IFN-γ (25). Similarly, in virologically suppressed HIV-1–infected patients, IL-32 transcript levels were significantly elevated in peripheral blood mononuclear cells and positively correlated with IFN-γ transcript levels (26). Taken together, IL-32γ, the most biologically active isoform of IL-32, may contribute to type II IFN responses, which are closely linked to disease activity in SLE, thereby providing a possible biological rationale for its potential as a serologic biomarker for assessing remission states in SLE. However, IL-12, IFN-γ, and IFN-related gene signatures were not assessed in the present study. Therefore, the proposed link between IL-32γ and type II IFN activity in SLE remains speculative and should not be interpreted as a definitive mechanism. Further mechanistic studies are required to clarify this relationship.

The potential influence of immunosuppressants should be considered when interpreting our findings. In a previous study of patients with atopic dermatitis, cyclosporine treatment was associated with a reduction in serum IL-32 levels (27), suggesting that immunosuppressants may influence circulating IL-32 levels. However, in our cohort, the use of individual immunosuppressants did not differ according to LLDAS, DORIS remission, and GC-free remission status (data not shown in the Results). These findings suggest that the effect of immunosuppressants on serum IL-32γ levels is less likely to have affected the observed associations between serum IL-32γ levels and disease states.

A wide range of biomarkers for diagnosis, disease activity, and organ manifestations has been investigated in SLE (28). With regard to SLE disease activity biomarkers, serum soluble CD137 levels (29), the long noncoding RNAs MIR31HG and NKILA (30), serum Nrf2 protein levels (31), and EPSTI1 expression (32) have recently been reported as potential biomarkers associated with disease activity. However, previous studies of disease activity biomarkers have primarily focused on their correlations with disease activity indices. In contrast, the present study specifically evaluated serum IL-32γ as a potential biomarker for remission assessment using clinically relevant treat-to-target endpoints, including LLDAS, DORIS remission, and GC-free remission. This remission-oriented approach highlights the distinct clinical relevance of IL-32γ among the other emerging biomarkers in SLE.

Some limitations should be acknowledged. First, the sample size was relatively small and included a single ethnic population, which may limit the generalizability of our findings. In addition, no independent external validation cohort was included, which may further limit the generalizability and robustness of our findings. As disease characteristics can vary across different ethnicities (33), replication in larger, multiethnic cohorts is warranted to confirm the robustness of IL-32γ as a biomarker. Owing to the relatively small sample size with only 8 and 7 patients in DORIS remission and GC-free remission, respectively, and the lack of an external validation cohort, the present findings should be interpreted as exploratory and hypothesis-generating rather than definitive. Second, this was a cross-sectional study, so longitudinal changes in serum IL-32γ levels and treatment-related effects could not be evaluated. In addition, temporal relationships between serum IL-32γ levels and subsequent clinical outcomes could not be established, and the ability of serum IL-32γ to predict remission, disease flare, or treatment response could not be assessed. Intra-patient fluctuations in serum IL-32γ levels during transitions between active disease, LLDAS, DORIS remission, and GC-free remission also could not be evaluated, limiting our ability to determine its utility as a longitudinal monitoring or predictive biomarker. Nonetheless, because the purpose of our study was to identify biomarkers indicative of the contemporaneous disease state rather than predictive markers of future outcomes, this design was appropriate for the study objective. Future longitudinal studies with serial measurements are warranted to determine whether serum IL-32γ can serve as a longitudinal monitoring or predictive biomarker. Third, mechanistic investigations were not performed, and thus the biological pathways linking IL-32γ to disease activity and remission remain speculative. However, given the exploratory nature of this study, these findings provide an initial framework, and future mechanistic studies are warranted to delineate the precise immunologic role of IL-32γ in SLE pathogenesis and remission.

## Conclusion

In conclusion, this study demonstrated that serum IL-32γ levels significantly correlated with disease activity in SLE. More importantly, by focusing on remission assessment, this study provides data suggesting a novel utility for serum IL-32γ. In particular, serum IL-32γ levels showed promising discriminatory ability for disease states such as LLDAS, DORIS remission, and GC-free remission in patients with SLE. Unlike conventional serologic markers, IL-32γ retained discriminative capacity even in deeper states of disease quiescence (i.e., DORIS remission and GC-free remission), underscoring its potential as a novel biomarker for remission detection. While further studies are required to validate these findings and elucidate the mechanistic basis, our results suggest that IL-32γ could complement existing tools in a treat-to-target strategy, providing clinicians with a simple serologic marker to guide therapy toward the ultimate goal of GC-free remission.

## Data Availability

The original contributions presented in the study are included in the article/supplementary material. Further inquiries can be directed to the corresponding authors.
